# Crystal structure of Sr_2_CdPt_2_ containing linear platinum chains

**DOI:** 10.1107/S2056989015024937

**Published:** 2016-01-09

**Authors:** Effendi Nawawi, Fakhili Gulo, Jürgen Köhler

**Affiliations:** a Department of Chemical Education, Sriwijaya University, Inderalaya, Ogan Ilir 30662, South Sumatra, Indonesia; b Max Planck Institut für Festkörperforschung, Heisenbergstr. 1, 70698 Stuttgart, Germany

**Keywords:** crystal structure, inter­metallic compound, linear platinum chains

## Abstract

The ternary inter­metallic title phase, distrontium cadmium diplatinum, was prepared from stoichiometric amounts of the elements at 1123 K for one day. The crystal structure adopts the ortho­rhom­bic Ca_2_GaCu_2_ structure type in space group *Immm*. Its main features are characterized by linear (Pt—Pt⋯Pt—Pt)_*n*_ chains that are aligned along [010] and condensed through cadmium atoms forming Cd-centred Pt_2_Cd_2/2_ rectangles to build up sheets parallel to (001). These sheets are connected to each other *via* alternating (001) sheets of strontium atoms along [001]. The strontium sheets consists of corrugated Sr_4_ units that are condensed to each other through edge-sharing parallel to [100].

## Abstract 2

The title compound, Sr_2_CdPt_2_, adopts the Ca_2_GaCu_2_ structure type and exhibits linear platinum chains with two different Pt—Pt bonds of 2.7341 (13) and 3.2010 (14) Å.

## Chemical context   

A large number of transition metal-based ternary inter­metallic phases have been studied in terms of metal–metal inter­actions and structure–property relationships (Corbett, 2010 ▸). Exploratory synthetic approaches in systems *A*/Cd/Pt (*A* = alkaline earth metal) have revealed a great compositional and structural diversity. The calcium phase Ca_6_Cd_16_Pt_8_ contains a three-dimensional array of isolated Cd_8_ tetra­hedral stars (TS) and a face-centred cube of Pt@Ca_6_ octa­hedra (Samal *et al.*, 2013 ▸) whilst the structure of Ca_6_Cd_11_Pt crystallizes in its own structure type consisting of apically inter­bonded Cd_7_ penta­gonal bipyramids and five-membered rings of Ca atoms (Gulo *et al.*, 2013 ▸). The strontium phase SrCd_4_Pt_2_ is made up of chains of edge-sharing Cd_4_ tetra­hedra bridged by four-bonded Sr atoms (Samal *et al.*, 2013 ▸) and SrCdPt presents six-membered rings of Sr atoms in a chair conformation (Gulo & Köhler, 2014 ▸). The barium phase BaCd_2_Pt exhibits zigzag chains of Ba atoms and Pt-centred boat and anti-boat conformations formed by six-membered rings of Cd atoms (Gulo & Köhler, 2015 ▸). The Pt-based ternary inter­metallic compounds with general formula *A*_2_ *X*Pt_2_ (*A* = alkaline-earth or rare-earth metal; *X* = diel, triel, or tetrel element) adopt five different structure types. Sr_2_InPt_2_ (Muts *et al.*, 2007 ▸) crystallizes in the monoclinic Ca_2_Ir_2_S type (Schoolaert & Jung, 2002 ▸), Pu_2_SnPt_2_ (Pereira *et al.*, 1997 ▸) in the tetra­gonal Mo_2_FeB_2_ type (Gladyshevskii *et al.*, 1996 ▸) while U_2_CdPt_2_ has its own structure type (Gravereau *et al.*, 1994 ▸). Ce_2_CdPt_2_ (Pöttgen *et al.*, 2000 ▸) adopts the tetra­gonal U_3_Si_2_ type (Zachariasen, 1948 ▸), and Ca_2_CdPt_2_ (Samal & Corbett, 2012 ▸) the ortho­rhom­bic Ca_2_GaCu_2_ type (Fornasini & Merlo, 1988 ▸).

In this article we present the crystal structure of the novel inter­metallic phase Sr_2_CdPt_2_ containing linear (Pt—Pt⋯Pt—Pt)_*n*_ chains as a principal structural motif.

## Structural commentary   

**Figure 1:**
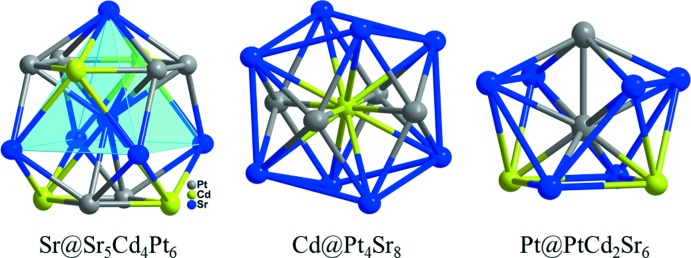
Coordination of strontium, cadmium, and platinum atoms in Sr_2_CdPt_2_. Displacement ellipsoids are displayed at the 90% probability level.

The ternary inter­metallic title phase adopts the ortho­rhom­bic Ca_2_GaCu_2_ structure type (Fornasini & Merlo, 1988 ▸) with the Ca, Ga, and Cu sites replaced by Sr, Cd, and Pt sites, respectively. The three atoms occupy three independent sites in the unit cell. The Sr atom resides on a special position with site symmetry *mm*2 (Wyckoff site 4 *j*), the Cd atom occupies a special positions with site symmetry *mmm* (2 *a*) and the Pt atom is on a special positions with site symmetry *m*2*m* (4 *h*). In the two structures, the transition metals (platinum and copper, respectively) occupy the same positions. In contrast, in the structure of SrCd_4_Pt_2_ (Samal *et al.*, 2013 ▸) which is isotypic with ZrFe_4_Si_2_ (Yarmolyuk *et al.*, 1975 ▸), the transition metals (platinum and iron, respectively) occupy different positions and the Pt atoms reside on the respective Si sites because silicon and platinum atoms are the most electronegative atoms in the two systems. The new phase Sr_2_CdPt_2_ contains 26 valence electrons and, as already mentioned, is isotypic with Ca_2_GaCu_2_. However, in comparison the structure of Sr_2_CdPt_2_ is appreciably distorted along the platinum chains, presumably because Ca_2_GaCu_2_ contains much smaller Cu atoms and a larger valence electron count of 29. Coordination spheres of each atomic site in the title structure are illustrated in Fig. 1 ▸. The Sr atom is coordinated by five other Sr, four Cd, and four Pt atoms. The Sr—Sr bond lengths vary from 3.674 (3) to 3.854 (1) Å, the Sr—Cd distances range from 3.490 (1) to 3.577 (1) Å, whereas the Sr—Pt values vary only slightly, from 3.188 (1) to 3.231 (1) Å. Six Sr atoms construct a square-planar pyramid, Sr@Sr_5_. The existence of Sr—Sr strong bonds is observable in SrCdPt (Gulo & Köhler, 2014 ▸) but not in SrCd_4_Pt_2_ (Samal *et al.*, 2013 ▸). The Cd atom exhibits a coordination number of twelve and has eight Sr and four Pt atoms in its environment with Cd—Pt distances of 2.785 (1) Å. The Pt atom is surrounded by six Sr, two Cd and one Pt atoms with a Pt—Pt distance of 2.734 (1) Å. This distance is slightly longer than those found in Ca_2_CdPt_2_ (2.659 Å; Samal & Corbett, 2012 ▸) or Sr_2_InPt_2_ (2.707 Å; Muts *et al.*, 2007 ▸) but shorter than those in Pu_2_SnPt_2_ (Pereira *et al.*, 1997 ▸), U_2_CdPt_2_ (Gravereau *et al.*, 1994 ▸) or Ce_2_CdPt_2_ (Pöttgen *et al.*, 2000 ▸). All other inter­atomic distances (Sr—Cd, Sr—Pt, and Cd—Pt) are in agreement with those found in some ternary compounds in *A*/Cd/Pt systems (*A* = alkaline earth metal).

## Packing features   

**Figure 2:**
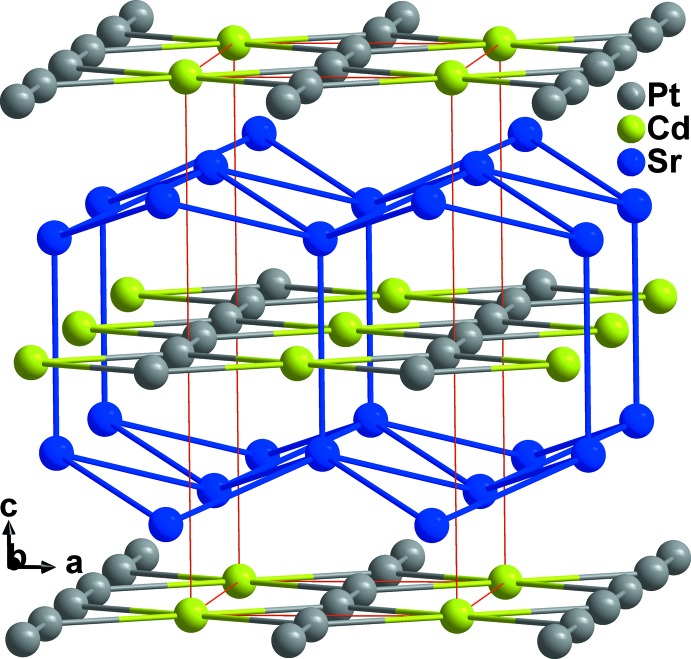
Projection of the crystal structure of Sr_2_CdPt_2_ approximately along the *b* axis.

**Figure 3:**
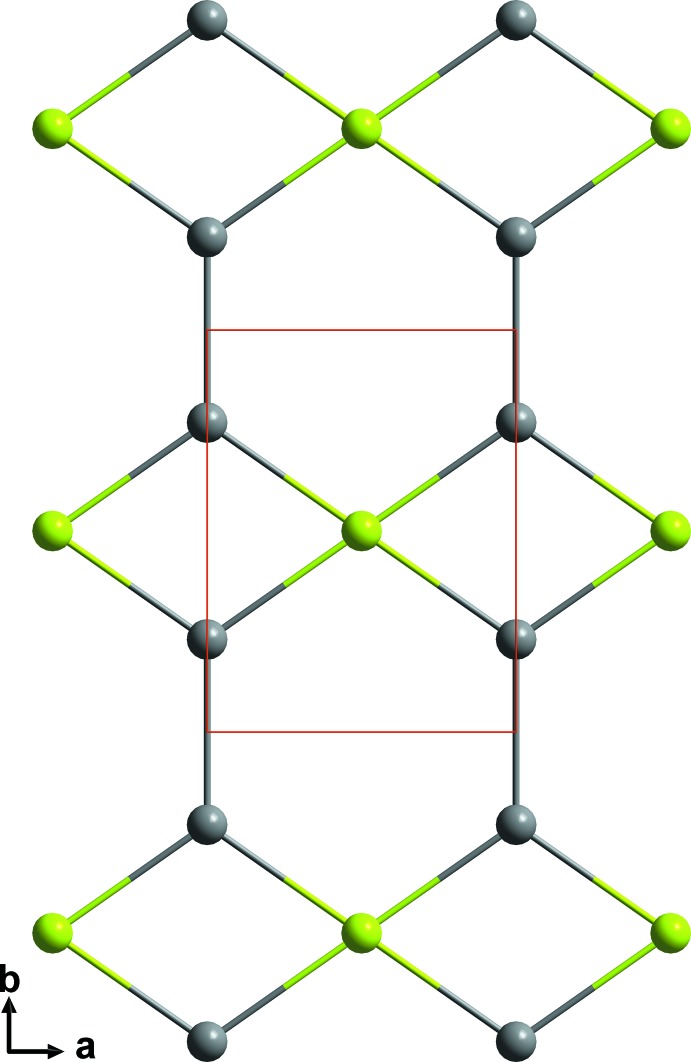
Projection of linear platinum chains that are aligned along the *b* axis and condensed *via* cadmium atoms forming Pt_2_Cd_2/2_-rectangles in the *ab*-plane

Sr atoms are bound together into corrugated sheets consisting of edge-sharing Sr_4_-units. These sheets spread parallel to (001) and are linked by another Sr—Sr bond of 3.674 (3) Å along [001]. (Fig. 2 ▸). The crystal structure is also characterized by the existence of linear (Pt—Pt⋯Pt—Pt)_*n*_ chains along [010] with longer distances of 3.2010 (14) Å between pairs of tightly bound Pt—Pt dumbbells, a significant distortion in the direction of dimerization. The platinum chains are condensed into (001) sheets through Cd atoms, forming Cd-centred rectangles with composition Pt_2_Cd_2/2_ (Fig. 3 ▸). The Pt_2_Cd_2/2_ layers are stacked along [001] and are linked through the corrugated sheets of Sr atoms.

## Database survey   

A search of the Pearson’s Crystal Data – Crystal Structure Database for Inorganic Compounds (Villars & Cenzual, 2015 ▸) for the Sr/Cd/Pt family of compounds returned two compounds only: SrCd_4_Pt_2_ (Samal *et al.*, 2013 ▸) and SrCdPt (Gulo & Köhler, 2014 ▸).

## Synthesis and crystallization   

The title compound was synthesized from starting materials of Sr granules (99.9+%, Alfa Aesar), Cd powder (99.9+%, Alfa Aesar) and Pt powder (99.95%, Chempur). A stoichiometric mixture of these elements was loaded into a Nb ampoule in an Ar-filled dry box. The Nb ampoule was then weld-sealed under an Ar atmosphere and subsequently enclosed in an evacuated silica jacket. The sample was then heated to 1123 K for 15 h, equilibrated at 923 K for 4 days, and followed by slow cooling to room temperature.

## Refinement   

Crystal data, data collection and structure refinement details are summarized in Table 1 ▸. The maximum and minimum remaining electron densities are located 1.66 and 0.81 Å, respectively, from the Pt site.

## Supplementary Material

Crystal structure: contains datablock(s) I. DOI: 10.1107/S2056989015024937/wm5254sup1.cif

Structure factors: contains datablock(s) I. DOI: 10.1107/S2056989015024937/wm5254Isup2.hkl

CCDC reference: 1444811

Additional supporting information:  crystallographic information; 3D view; checkCIF report

## Figures and Tables

**Table 1:** Experimental details

Crystal data	
Chemical formula	CdPt_2_Sr_2_
*M*_r_	677.82
Crystal system, space group	Orthorhombic, *I* *m* *m* *m*
Temperature (K)	293
*a*, *b*, *c* (Å)	4.5596 (9), 5.9351 (12), 9.1874 (18)
*V* (Å^3^)	248.63 (9)
*Z*	2
Radiation type	Mo *K*α
μ (mm^−1^)	81.39
Crystal size (mm)	0.08 × 0.06 × 0.05

Data collection	
Diffractometer	Bruker P4
Absorption correction	Multi-scan (*SADABS*; Bruker, 2001 ▸)
*T*_min_, *T*_max_	0.004, 0.017
No. of measured, independent and observed [*I* > 2σ(*I*)] reflections	1069, 190, 172
*R*_int_	0.025
(sin θ/λ)_max_ (Å^−1^)	0.666

Refinement	
*R*[*F* ^2^ > 2σ(*F* ^2^)], *wR*(*F* ^2^), *S*	0.022, 0.058, 1.16
No. of reflections	190
No. of parameters	13
Δρ_max_, Δρ_min_ (e Å^−3^)	1.84, −2.51

Computer programs: *XSCANS* (Bruker, 2001 ▸), *SHELXS97* and *SHELXL97* (Sheldrick, 2008 ▸) and *DIAMOND* (Brandenburg, 2010 ▸).

## supplementary crystallographic information

### Crystal data

**Table:**

CdPt_2_Sr_2_	*F*(000) = 560
*M**_r_* = 677.82	*D*_x_ = 9.054 Mg m^−^^3^
Orthorhombic, *I**m**m**m*	Mo *K*α radiation, λ = 0.71073 Å
Hall symbol: -I 2 2	Cell parameters from 25 reflections
*a* = 4.5596 (9) Å	θ = 12–18°
*b* = 5.9351 (12) Å	µ = 81.39 mm^−^^1^
*c* = 9.1874 (18) Å	*T* = 293 K
*V* = 248.63 (9) Å^3^	Block, brown
*Z* = 2	0.08 × 0.06 × 0.05 mm

### Data collection

**Table:**

Bruker P4 4-circle diffractometer	190 independent reflections
Radiation source: fine-focus sealed tube	172 reflections with *I* > 2σ(*I*)
Graphite monochromator	*R*_int_ = 0.025
Detector resolution: 0 pixels mm^-1^	θ_max_ = 28.3°, θ_min_ = 4.1°
ω–scans	*h* = −5→6
Absorption correction: multi-scan (*SADABS*; Bruker, 2001)	*k* = −7→7
*T*_min_ = 0.004, *T*_max_ = 0.017	*l* = −11→11
1069 measured reflections	

### Refinement

**Table:**

Refinement on *F*^2^	Primary atom site location: structure-invariant direct methods
Least-squares matrix: full	Secondary atom site location: difference Fourier map
*R*[*F*^2^ > 2σ(*F*^2^)] = 0.022	*w* = 1/[σ^2^(*F*_o_^2^) + (0.0337*P*)^2^ + 3.9776*P*] where *P* = (*F*_o_^2^ + 2*F*_c_^2^)/3
*wR*(*F*^2^) = 0.058	(Δ/σ)_max_ < 0.001
*S* = 1.16	Δρ_max_ = 1.84 e Å^−^^3^
190 reflections	Δρ_min_ = −2.51 e Å^−^^3^
13 parameters	Extinction correction: *SHELXL*, Fc^*^=kFc[1+0.001xFc^2^λ^3^/sin(2θ)]^-1/4^
0 restraints	Extinction coefficient: 0.0024 (4)

### Special details

**Table:**

Geometry. All e.s.d.'s (except the e.s.d. in the dihedral angle between two l.s. planes) are estimated using the full covariance matrix. The cell e.s.d.'s are taken into account individually in the estimation of e.s.d.'s in distances, angles and torsion angles; correlations between e.s.d.'s in cell parameters are only used when they are defined by crystal symmetry. An approximate (isotropic) treatment of cell e.s.d.'s is used for estimating e.s.d.'s involving l.s. planes.
Refinement. Refinement of *F*^2^ against ALL reflections. The weighted *R*-factor *wR* and goodness of fit *S* are based on *F*^2^, conventional *R*-factors *R* are based on *F*, with *F* set to zero for negative *F*^2^. The threshold expression of *F*^2^ > σ(*F*^2^) is used only for calculating *R*-factors(gt) *etc*. and is not relevant to the choice of reflections for refinement. *R*-factors based on *F*^2^ are statistically about twice as large as those based on *F*, and *R*- factors based on ALL data will be even larger.

### Fractional atomic coordinates and isotropic or equivalent isotropic displacement parameters (Å^2^)

**Table:**

	*x*	*y*	*z*	*U*_iso_*/*U*_eq_	
Pt	0.0000	0.23034 (10)	0.5000	0.0172 (3)	
Cd	−0.5000	0.5000	0.5000	0.0172 (4)	
Sr	0.0000	0.5000	0.19995 (16)	0.0162 (4)	

### Atomic displacement parameters (Å^2^)

**Table:**

	*U*^11^	*U*^22^	*U*^33^	*U*^12^	*U*^13^	*U*^23^
Pt	0.0156 (4)	0.0205 (4)	0.0155 (4)	0.000	0.000	0.000
Cd	0.0157 (8)	0.0152 (8)	0.0207 (9)	0.000	0.000	0.000
Sr	0.0171 (8)	0.0169 (8)	0.0146 (8)	0.000	0.000	0.000

### Geometric parameters (Å, º)

**Table:**

Pt—Pt^i^	2.7341 (13)	Cd—Sr^vii^	3.4901 (10)
Pt—Cd^ii^	2.7855 (5)	Cd—Sr^viii^	3.5773 (12)
Pt—Cd	2.7855 (5)	Cd—Sr^iii^	3.5773 (13)
Pt—Sr^iii^	3.1876 (14)	Cd—Sr	3.5773 (12)
Pt—Sr	3.1876 (14)	Cd—Sr^ix^	3.5773 (13)
Pt—Pt^iii^	3.2010 (14)	Sr—Pt^iii^	3.1876 (14)
Pt—Sr^iv^	3.2312 (10)	Sr—Pt^iv^	3.2312 (10)
Pt—Sr^v^	3.2312 (10)	Sr—Pt^xii^	3.2312 (10)
Pt—Sr^vi^	3.2312 (10)	Sr—Pt^xiii^	3.2312 (10)
Pt—Sr^vii^	3.2312 (10)	Sr—Pt^v^	3.2312 (10)
Cd—Pt^iii^	2.7855 (5)	Sr—Cd^xiv^	3.4901 (10)
Cd—Pt^viii^	2.7855 (5)	Sr—Cd^xiii^	3.4901 (10)
Cd—Pt^ix^	2.7855 (5)	Sr—Cd^ii^	3.5773 (12)
Cd—Sr^iv^	3.4901 (10)	Sr—Sr^xv^	3.674 (3)
Cd—Sr^x^	3.4901 (10)	Sr—Sr^xvi^	3.8535 (9)
Cd—Sr^xi^	3.4901 (10)		
			
Pt^i^—Pt—Cd^ii^	125.070 (13)	Sr^viii^—Cd—Sr^iii^	180.0
Pt^i^—Pt—Cd	125.070 (13)	Pt^iii^—Cd—Sr	58.560 (14)
Cd^ii^—Pt—Cd	109.86 (3)	Pt^viii^—Cd—Sr	121.440 (14)
Pt^i^—Pt—Sr^iii^	120.139 (18)	Pt^ix^—Cd—Sr	121.440 (14)
Cd^ii^—Pt—Sr^iii^	73.232 (13)	Pt—Cd—Sr	58.560 (14)
Cd—Pt—Sr^iii^	73.232 (13)	Sr^iv^—Cd—Sr	66.071 (12)
Pt^i^—Pt—Sr	120.139 (18)	Sr^x^—Cd—Sr	113.929 (12)
Cd^ii^—Pt—Sr	73.232 (13)	Sr^xi^—Cd—Sr	66.071 (12)
Cd—Pt—Sr	73.232 (13)	Sr^vii^—Cd—Sr	113.929 (12)
Sr^iii^—Pt—Sr	119.72 (4)	Sr^viii^—Cd—Sr	79.18 (3)
Pt^i^—Pt—Pt^iii^	180.0	Sr^iii^—Cd—Sr	100.82 (3)
Cd^ii^—Pt—Pt^iii^	54.930 (13)	Pt^iii^—Cd—Sr^ix^	121.440 (14)
Cd—Pt—Pt^iii^	54.930 (13)	Pt^viii^—Cd—Sr^ix^	58.560 (14)
Sr^iii^—Pt—Pt^iii^	59.861 (18)	Pt^ix^—Cd—Sr^ix^	58.560 (14)
Sr—Pt—Pt^iii^	59.861 (18)	Pt—Cd—Sr^ix^	121.440 (14)
Pt^i^—Pt—Sr^iv^	64.971 (13)	Sr^iv^—Cd—Sr^ix^	113.929 (12)
Cd^ii^—Pt—Sr^iv^	145.14 (2)	Sr^x^—Cd—Sr^ix^	66.071 (12)
Cd—Pt—Sr^iv^	70.466 (13)	Sr^xi^—Cd—Sr^ix^	113.929 (12)
Sr^iii^—Pt—Sr^iv^	134.76 (2)	Sr^vii^—Cd—Sr^ix^	66.071 (12)
Sr—Pt—Sr^iv^	73.786 (15)	Sr^viii^—Cd—Sr^ix^	100.82 (3)
Pt^iii^—Pt—Sr^iv^	115.029 (13)	Sr^iii^—Cd—Sr^ix^	79.18 (3)
Pt^i^—Pt—Sr^v^	64.971 (13)	Sr—Cd—Sr^ix^	180.0
Cd^ii^—Pt—Sr^v^	70.466 (13)	Pt^iii^—Sr—Pt	60.28 (4)
Cd—Pt—Sr^v^	145.14 (2)	Pt^iii^—Sr—Pt^iv^	134.76 (2)
Sr^iii^—Pt—Sr^v^	134.76 (2)	Pt—Sr—Pt^iv^	106.214 (15)
Sr—Pt—Sr^v^	73.786 (15)	Pt^iii^—Sr—Pt^xii^	106.214 (15)
Pt^iii^—Pt—Sr^v^	115.029 (13)	Pt—Sr—Pt^xii^	134.76 (2)
Sr^iv^—Pt—Sr^v^	89.75 (3)	Pt^iv^—Sr—Pt^xii^	50.06 (3)
Pt^i^—Pt—Sr^vi^	64.971 (13)	Pt^iii^—Sr—Pt^xiii^	106.214 (15)
Cd^ii^—Pt—Sr^vi^	70.466 (13)	Pt—Sr—Pt^xiii^	134.76 (2)
Cd—Pt—Sr^vi^	145.14 (2)	Pt^iv^—Sr—Pt^xiii^	110.71 (5)
Sr^iii^—Pt—Sr^vi^	73.786 (15)	Pt^xii^—Sr—Pt^xiii^	89.75 (3)
Sr—Pt—Sr^vi^	134.76 (2)	Pt^iii^—Sr—Pt^v^	134.76 (2)
Pt^iii^—Pt—Sr^vi^	115.029 (13)	Pt—Sr—Pt^v^	106.214 (15)
Sr^iv^—Pt—Sr^vi^	129.94 (3)	Pt^iv^—Sr—Pt^v^	89.75 (3)
Sr^v^—Pt—Sr^vi^	69.29 (5)	Pt^xii^—Sr—Pt^v^	110.71 (5)
Pt^i^—Pt—Sr^vii^	64.971 (13)	Pt^xiii^—Sr—Pt^v^	50.06 (3)
Cd^ii^—Pt—Sr^vii^	145.14 (2)	Pt^iii^—Sr—Cd^xiv^	151.90 (4)
Cd—Pt—Sr^vii^	70.466 (13)	Pt—Sr—Cd^xiv^	91.620 (18)
Sr^iii^—Pt—Sr^vii^	73.786 (15)	Pt^iv^—Sr—Cd^xiv^	48.779 (16)
Sr—Pt—Sr^vii^	134.76 (2)	Pt^xii^—Sr—Cd^xiv^	93.47 (3)
Pt^iii^—Pt—Sr^vii^	115.029 (13)	Pt^xiii^—Sr—Cd^xiv^	93.47 (3)
Sr^iv^—Pt—Sr^vii^	69.29 (5)	Pt^v^—Sr—Cd^xiv^	48.779 (16)
Sr^v^—Pt—Sr^vii^	129.94 (3)	Pt^iii^—Sr—Cd^xiii^	91.620 (19)
Sr^vi^—Pt—Sr^vii^	89.75 (3)	Pt—Sr—Cd^xiii^	151.90 (4)
Pt^iii^—Cd—Pt^viii^	180.0	Pt^iv^—Sr—Cd^xiii^	93.47 (3)
Pt^iii^—Cd—Pt^ix^	109.86 (3)	Pt^xii^—Sr—Cd^xiii^	48.779 (16)
Pt^viii^—Cd—Pt^ix^	70.14 (3)	Pt^xiii^—Sr—Cd^xiii^	48.779 (16)
Pt^iii^—Cd—Pt	70.14 (3)	Pt^v^—Sr—Cd^xiii^	93.47 (3)
Pt^viii^—Cd—Pt	109.86 (3)	Cd^xiv^—Sr—Cd^xiii^	116.48 (4)
Pt^ix^—Cd—Pt	180.0	Pt^iii^—Sr—Cd^ii^	48.21 (2)
Pt^iii^—Cd—Sr^iv^	119.245 (13)	Pt—Sr—Cd^ii^	48.21 (2)
Pt^viii^—Cd—Sr^iv^	60.755 (13)	Pt^iv^—Sr—Cd^ii^	152.59 (2)
Pt^ix^—Cd—Sr^iv^	119.245 (13)	Pt^xii^—Sr—Cd^ii^	152.59 (2)
Pt—Cd—Sr^iv^	60.755 (13)	Pt^xiii^—Sr—Cd^ii^	89.339 (16)
Pt^iii^—Cd—Sr^x^	60.755 (13)	Pt^v^—Sr—Cd^ii^	89.339 (16)
Pt^viii^—Cd—Sr^x^	119.245 (13)	Cd^xiv^—Sr—Cd^ii^	113.929 (12)
Pt^ix^—Cd—Sr^x^	60.755 (13)	Cd^xiii^—Sr—Cd^ii^	113.929 (12)
Pt—Cd—Sr^x^	119.245 (13)	Pt^iii^—Sr—Cd	48.21 (2)
Sr^iv^—Cd—Sr^x^	180.00 (4)	Pt—Sr—Cd	48.21 (2)
Pt^iii^—Cd—Sr^xi^	60.755 (13)	Pt^iv^—Sr—Cd	89.339 (16)
Pt^viii^—Cd—Sr^xi^	119.245 (13)	Pt^xii^—Sr—Cd	89.339 (16)
Pt^ix^—Cd—Sr^xi^	60.755 (13)	Pt^xiii^—Sr—Cd	152.59 (2)
Pt—Cd—Sr^xi^	119.245 (13)	Pt^v^—Sr—Cd	152.59 (2)
Sr^iv^—Cd—Sr^xi^	116.48 (4)	Cd^xiv^—Sr—Cd	113.929 (12)
Sr^x^—Cd—Sr^xi^	63.52 (4)	Cd^xiii^—Sr—Cd	113.929 (12)
Pt^iii^—Cd—Sr^vii^	119.245 (13)	Cd^ii^—Sr—Cd	79.18 (3)
Pt^viii^—Cd—Sr^vii^	60.755 (13)	Pt^iii^—Sr—Sr^xv^	149.861 (18)
Pt^ix^—Cd—Sr^vii^	119.245 (13)	Pt—Sr—Sr^xv^	149.861 (18)
Pt—Cd—Sr^vii^	60.755 (13)	Pt^iv^—Sr—Sr^xv^	55.35 (2)
Sr^iv^—Cd—Sr^vii^	63.52 (4)	Pt^xii^—Sr—Sr^xv^	55.35 (2)
Sr^x^—Cd—Sr^vii^	116.48 (4)	Pt^xiii^—Sr—Sr^xv^	55.35 (2)
Sr^xi^—Cd—Sr^vii^	180.0	Pt^v^—Sr—Sr^xv^	55.35 (2)
Pt^iii^—Cd—Sr^viii^	121.440 (14)	Cd^xiv^—Sr—Sr^xv^	58.24 (2)
Pt^viii^—Cd—Sr^viii^	58.560 (14)	Cd^xiii^—Sr—Sr^xv^	58.24 (2)
Pt^ix^—Cd—Sr^viii^	58.560 (14)	Cd^ii^—Sr—Sr^xv^	140.409 (17)
Pt—Cd—Sr^viii^	121.440 (14)	Cd—Sr—Sr^xv^	140.409 (17)
Sr^iv^—Cd—Sr^viii^	66.071 (12)	Pt^iii^—Sr—Sr^xvi^	53.63 (3)
Sr^x^—Cd—Sr^viii^	113.929 (12)	Pt—Sr—Sr^xvi^	100.38 (5)
Sr^xi^—Cd—Sr^viii^	66.071 (12)	Pt^iv^—Sr—Sr^xvi^	151.51 (5)
Sr^vii^—Cd—Sr^viii^	113.929 (12)	Pt^xii^—Sr—Sr^xvi^	103.14 (3)
Pt^iii^—Cd—Sr^iii^	58.560 (14)	Pt^xiii^—Sr—Sr^xvi^	52.59 (2)
Pt^viii^—Cd—Sr^iii^	121.440 (14)	Pt^v^—Sr—Sr^xvi^	92.53 (2)
Pt^ix^—Cd—Sr^iii^	121.440 (14)	Cd^xiv^—Sr—Sr^xvi^	141.30 (3)
Pt—Cd—Sr^iii^	58.560 (14)	Cd^xiii^—Sr—Sr^xvi^	58.05 (2)
Sr^iv^—Cd—Sr^iii^	113.929 (12)	Cd^ii^—Sr—Sr^xvi^	55.88 (3)
Sr^x^—Cd—Sr^iii^	66.071 (12)	Cd—Sr—Sr^xvi^	101.13 (5)
Sr^xi^—Cd—Sr^iii^	113.929 (12)	Sr^xv^—Sr—Sr^xvi^	103.81 (4)
Sr^vii^—Cd—Sr^iii^	66.071 (12)		

Symmetry codes: (i) −*x*, −*y*, −*z*+1; (ii) *x*+1, *y*, *z*; (iii) −*x*, −*y*+1, −*z*+1; (iv) −*x*−1/2, −*y*+1/2, −*z*+1/2; (v) −*x*+1/2, −*y*+1/2, −*z*+1/2; (vi) *x*+1/2, *y*−1/2, *z*+1/2; (vii) *x*−1/2, *y*−1/2, *z*+1/2; (viii) *x*−1, *y*, *z*; (ix) −*x*−1, −*y*+1, −*z*+1; (x) *x*−1/2, *y*+1/2, *z*+1/2; (xi) −*x*−1/2, −*y*+3/2, −*z*+1/2; (xii) *x*−1/2, *y*+1/2, *z*−1/2; (xiii) *x*+1/2, *y*+1/2, *z*−1/2; (xiv) *x*+1/2, *y*−1/2, *z*−1/2; (xv) −*x*, −*y*+1, −*z*; (xvi) −*x*+1/2, −*y*+3/2, −*z*+1/2.
